# Supplementary material for: Spinal neural tube formation and tail development in human embryos
Source: eLife. 2024 Dec 5;12:RP88584. doi: 10.7554/eLife.88584 (PMC11620743; doi:10.7554/eLife.88584)
Supplement: Supplementary file 1. — Rows show individual publications; columns show the topics covered in publications. [file elife-88584-supp1.docx]

**Supplementary File 1. Publications on human secondary neural tube and body formation, showing topics covered**

| **Study** | **Samples** | **PNP closure** | **Primary to secondary NT transition** | **Mode of secondary NT formation** | **Multiple NT lumens observed** | **Formation of secondary somites, notochord, tail-gut** | **Morphology of tail regression** | **Mode of cell death during regression** | **Gene expression** |
| --- | --- | --- | --- | --- | --- | --- | --- | --- | --- |
| **Kunitomo** (1918). Carnegie Inst. Contr. Embryol 8, 161-198. | 44 embryos, 4-125 mm crown-rump length, sections analysed from Carnegie collection |  | X | X | X |  | X |  |  |
| **Streeter** (1919). Am. J. Anat. 25, 1-11. | Unspecified number of embryos, sections analysed from Carnegie collection |  |  | X | X |  | X |  |  |
| **Bolli** (1966). Acta. Anat. 64, 48-81. | 123 normal embryos, 2-150 mm CRL, with unspecified method of collection |  |  | X | X |  | X |  |  |
| **Lemire** (1969). Teratology 2, 361-370. | 8 embryos of CRL 6-25 mm, CS14-21, including from spontaneous abortions (3) and ectopic pregnancies (4) |  |  | X | X |  | X | X |  |
| **Hughes** et al. (1974). J. Embryol. Exp. Morphol. 32, 355-363. | 6 embryos at 28-40 days, sections analysed, with unspecified method of collection |  |  | X | X |  |  |  |  |
| **Fallon** et al. (1978). Am. J. Anat. 152, 111-129. | 52 embryos at CS14-22 from induced abortion |  |  | X | X |  | X | X |  |
| **Muller** et al. (1987). Anat. Embryol. 176, 413-430. | 24 embryos at CS12, sections analysed from Carnegie collection | X | X | X |  | X |  |  |  |
| **Muller** et al. (1988). Anat. Embryol. (Berl) 177, 203-224. | 25 embryos at CS13, sections analysed from Carnegie collection |  |  | X | X | X |  |  |  |
| **Nievelstein** et al. (1993). Teratology 48, 21-31. | 36 embryos CS11-23 of unspecified origin | X | X | X |  |  | X | X |  |
| **Saraga-Babic** et al. (1994). Ann. Anat. 176, 277-286. | 20 embryos at 4-12 weeks, obtained from artificial abortions |  |  | X |  | X | X |  | X |
| **Saraga-Babic** et al. (1995). J. Brain Res. 36, 341-347. | 15 embryos aged 4-8 developmental weeks (CS12-23) from artificial abortions | X | X | X | X |  |  |  |  |
| **Peeters** et al. (1998). Anat. Embryol. 198, 185-194. | 11 embryos CS10-12, photographs analysed, of Kyto embryos from induced abortion for social reasons | X |  |  |  |  |  |  |  |
| **Nakatsu** et al. (2000). Anat. Embryol. (Berl) 201, 455-466. | 68 normal embryos CS10-12 from pregnancy terminations for social reasons; a few from spontaneous or emergency abortions | X |  |  |  |  |  |  |  |
| **Sapunar** et al. (2001). Ann. Anat. 183, 217-222. | 8 embryos at CS12-18 obtained from artificial abortions |  |  |  |  |  | X | X |  |
| **O'Rahilly** et al. (2002). Teratology 65, 162-170. | 98 embryos at CS8-13, sections analysed from Carnegie collection | X |  |  |  |  |  |  |  |
| **Muller** et al. (2004). Cells Tissues Organs 177, 2-20. | 52 embryos at CS9-23, sections analysed from Carnegie collection | X | X | X | X | X | X |  |  |
| **Saitsu** et al. (2004). Anat. Embryol. (Berl) 209, 107-117. | 20 embryos around stage of posterior neuropore closure (CS12-13). Most from induced abortion for social reasons; one from ectopic pregnancy | X | X | X | X |  |  |  |  |
| **Vilovic** et al. (2006). Anat. Embryol. 211, 1-9. | 18 embryos at CS12-CS23 (5-12 weeks), from spontaneous or induced abortions |  |  |  |  |  | X | X | X |
| **Pytel** et al. (2007). Folia Morphol. (Warsz. ) 66, 104-108. | 12 embryos at CS13-17, 32-41 days, with unspecified method of collection |  |  | X | X |  |  |  |  |
| **Saitsu** et al. (2008). Congenit. Anom. (Kyoto) 48, 1-6. | 43 embryos obtained from Kyoto collection from induced abortion for social reasons | X | X | X | X |  |  |  |  |
| **Krupp** et al. (2012). Birth Defects Res. A Clin. Mol. Teratol. 94, 683-692. | 8 or more CS12 and CS13 embryos, from mifepristone induced abortions |  |  |  |  |  |  |  | X |
| **Olivera-Martinez** et al. (2012). PLoS Biol .10, e1001415. | 3 embryos at CS12 (2) and CS16 (1) from induced abortion for social reasons |  |  |  | X |  |  |  | X |
| **Yang** et al. (2014). Childs Nerv. Syst. 30, 73-82. | 21 embryos at CS12-23, from therapeutic pregnancy termination or surgical procedures to remove uterus/oviduct |  |  | X | X | X | X | X | X |
| **Jang** et al. (2016). Pediatr. Neurosurg. 51, 9-19. | 20 embryos and fetuses, 6-14 weeks gestation from miscarriages and ectopic pregnancies |  | X |  |  |  |  |  |  |
| **Tojima** et al. (2018). J. Anat. 232, 806-811. | 42 embryos at CS13-23 from Kyoto collection from induced abortion for social reasons |  |  |  |  | X | X |  |  |

Abbreviations: CS, Carnegie Stage; NT, neural tube; PNP, posterior neuropore
