# Supplementary material for: Spinal neural tube formation and tail development in human embryos
Source: eLife. 2024 Dec 5;12:RP88584. doi: 10.7554/eLife.88584 (PMC11620743; doi:10.7554/eLife.88584)
Supplement: Supplementary file 2. — Each line in the table corresponds to a different human embryo (n=37). Measurements of crown-rump length, tail length, and tail length distal to somites were not available for all embryos. Data in Figure 2I–K and Table 2 are based on the available data. [file elife-88584-supp2.docx]

**Supplementary File 2. Source data on human embryos as summarised in Figure 2I-K and Table 2 ***

| **Carnegie Stage** | **Days pc** | **Somite no.** | **Crown-rump length (mm) **** | **Tail length (mm) **** | **Tail length distal to somites (mm) **** |
| --- | --- | --- | --- | --- | --- |
| 13 | 28 | 37 | 7.00 | 0.80 |  |
|  | 28 | 38 | 5.33 | 0.78 | 0.29 |
|  | 28 | 34 | 4.66 | 0.69 | 0.50 |
|  | 29 | 35 | 6.58 | 0.63 | 0.38 |
|  | 30 | 38 | 6.88 | 1.75 | 0.52 |
|  | 30 | 34 | 8.16 | 1.30 | 0.47 |
|  | 30 | 32 |  | 1.45 | 0.76 |
| 14 | 31 | 36 |  |  |  |
|  | 31 | 32 |  |  |  |
|  | 31 | 36 | 8.70 | 1.15 | 0.60 |
|  | 31 | 37 | 6.00 | 1.08 | 0.52 |
|  | 32 | 34 | 10.38 | 0.75 |  |
| 15 | 33 | 37 | 9.00 | 1.20 | 0.56 |
|  | 33 | 34 | 8.60 | 2.00 | 0.91 |
|  | 34 | 37 | 9.75 | 1.48 | 0.60 |
|  | 34 | 38 | 7.80 |  | 0.78 |
|  | 35 | 37 | 9.38 | 1.25 | 0.60 |
|  | 35 | 34 |  | 0.50 | 0.54 |
|  | 35 | 34 |  | 0.65 |  |
| 16 | 37 | 39 |  | 1.10 |  |
|  | 37 | 39 |  | 1.05 | 0.35 |
|  | 37 | 33 | 12.33 | 1.19 | 0.38 |
|  | 37 | 39 |  | 0.95 |  |
|  | 37 | 35 | 12.50 | 2.44 | 0.64 |
|  | 38 | 38 | 10.13 | 1.08 |  |
|  | 38 | 37 | 10.66 | 1.20 | 0.46 |
|  | 39 | 33 | 12.75 | 1.33 |  |
| 17 | 40 | 34 | 10.88 | 1.44 | 0.35 |
|  | 40 | 34 | 13.25 | 1.25 |  |
|  | 42 | 31 |  | 1.05 | 0.33 |
|  | 43 | 30 | 12.50 | 1.00 |  |
| 18 | 44 |  |  |  |  |
|  | 44 | 33 | 15.88 | 1.25 | 0.00 |
|  | 45 | 31 | 18.13 | 0.63 |  |
|  | 45 | 34 | 14.50 | 1.38 |  |
|  | 45 | 33 | 13.00 | 1.76 |  |
|  | 45 | 32 |  | 0.70 |  |

* Each line in the table corresponds to a different human embryo (n = 37).

** Measurements of crown-rump length, tail length and tail length distal to somites were not available for all embryos. Data in Figure 2I-K and Table 2 are based on the available data.
